# IOP induces upregulation of GFAP and MHC-II and microglia reactivity in mice retina contralateral to experimental glaucoma

**DOI:** 10.1186/1742-2094-9-92

**Published:** 2012-05-14

**Authors:** Beatriz I Gallego, Juan J Salazar, Rosa de Hoz, Blanca Rojas, Ana I Ramírez, Manuel Salinas-Navarro, Arturo Ortín-Martínez, Francisco J Valiente-Soriano, Marcelino Avilés-Trigueros, Maria P Villegas-Perez, Manuel Vidal-Sanz, Alberto Triviño, Jose M Ramírez

**Affiliations:** 1 Instituto de Investigaciones Oftalmológicas Ramón Castroviejo, Universidad Complutense de Madrid, Madrid, 28040, Spain; 2 Escuela Universitaria de Óptica, Universidad Complutense de Madrid, Madrid, 28037, Spain; 3 Departamento de Oftalmología, Facultad de Medicina, Universidad Complutense de Madrid, Madrid, 28040, Spain; 4 Department of Ophthalmology, School of Medicine, Campus Universitario de Espinardo, Murcia University, Murcia, Espinardo, 30100, Spain

**Keywords:** Experimental glaucoma, Mice, Microglia, Astrocytes, Müller cell, Retina, GFAP, MHC-II

## Abstract

**Background:**

Ocular hypertension is a major risk factor for glaucoma, a neurodegenerative disease characterized by an irreversible decrease in ganglion cells and their axons. Macroglial and microglial cells appear to play an important role in the pathogenic mechanisms of the disease. Here, we study the effects of laser-induced ocular hypertension (OHT) in the macroglia, microglia and retinal ganglion cells (RGCs) of eyes with OHT (OHT-eyes) and contralateral eyes two weeks after lasering.

**Methods:**

Two groups of adult Swiss mice were used: age-matched control (naïve, n = 9); and lasered (n = 9). In the lasered animals, both OHT-eyes and contralateral eyes were analyzed. Retinal whole-mounts were immunostained with antibodies against glial fibrillary acid protein (GFAP), neurofilament of 200kD (NF-200), ionized calcium binding adaptor molecule (Iba-1) and major histocompatibility complex class II molecule (MHC-II). The GFAP-labeled retinal area (GFAP-RA), the intensity of GFAP immunoreaction (GFAP-IR), and the number of astrocytes and NF-200 + RGCs were quantified.

**Results:**

In comparison with naïve: i) astrocytes were more robust in contralateral eyes. In OHT-eyes, the astrocyte population was not homogeneous, given that astrocytes displaying only primary processes coexisted with astrocytes in which primary and secondary processes could be recognized, the former having less intense GFAP-IR (*P* < 0.001); ii) GFAP-RA was increased in contralateral (*P* <0.05) and decreased in OHT-eyes (*P* <0.001); iii) the mean intensity of GFAP-IR was higher in OHT-eyes (*P* < 0.01), and the percentage of the retinal area occupied by GFAP+ cells with higher intensity levels was increased in contralateral (*P* = 0.05) and in OHT-eyes (*P* < 0.01); iv) both in contralateral and in OHT-eyes, GFAP was upregulated in Müller cells and microglia was activated; v) MHC-II was upregulated on macroglia and microglia. In microglia, it was similarly expressed in contralateral and OHT-eyes. By contrast, in macroglia, MHC-II upregulation was observed mainly in astrocytes in contralateral eyes and in Müller cells in OHT-eyes; vi) NF-200+RGCs (degenerated cells) appeared in OHT-eyes with a trend for the GFAP-RA to decrease and for the NF-200+RGC number to increase from the center to the periphery (r = −0.45).

**Conclusion:**

The use of the contralateral eye as an internal control in experimental induction of unilateral IOP should be reconsidered. The gliotic behavior in contralateral eyes could be related to the immune response. The absence of NF-200+RGCs (sign of RGC degeneration) leads us to postulate that the MHC-II upregulation in contralateral eyes could favor neuroprotection.

## Background

Ocular hypertension is a major risk factor for glaucoma, a neurodegenerative disease characterized by an irreversible decrease of ganglion cells and their axons, the functional impact of which leads to a visual-field loss [1-7].

Although the hypothesis is generally accepted that glaucomatous damage is a consequence of axonal degeneration that ends with the death of ganglion cells, recent studies have shown the important role played by glia in the pathogenic mechanism of the disease [8-12].

Under normal conditions, astrocytes and Müller glia make contact with retinal neurons, providing stability to the neural tissue [13]. Physiological studies have demonstrated that both cell populations perform equivalent functions, including: storing glycogen, providing glucose to neurons, regulating the levels of extracellular potassium, playing a major role in the regulation and metabolism of neurotransmitters such as GABA, helping to remove CO_2_ from the retina, and contributing to the maintenance of water homeostasis in the retina [12,14-18]. Furthermore, astrocytes as well as Müller cells can induce blood–brain barrier properties within the vascular endothelial cells [19].

Resident glia in the retina and optic-nerve head alter their gene-expression profile during activation, presumably exerting neuroprotective or damaging influences at different phases of disease progression [20]. In the glaucomatous optic neuropathy, glial cells from the retina and from the optic nerve show abnormal behavior. This results in the expression of glial fibrillary acid protein (GFAP) in Müller glia and the appearance of reactive astrocytes, which are characterized by a change in their form and their GFAP expression [21].

The neurofilaments from retinal ganglion cells (RGCs) undergo alterations in glaucoma. It has been reported that the excitatory neurotransmitter glutamate, which exhibits elevated extracellular levels in pathologies such as glaucoma, can enhance the phosphorylation of neurofilaments [22] and induce the accumulation of neurofilaments in the neuronal soma [23]. Additionally, the interference of axonal transport has been proposed as one possible mechanism of neurofilament-induced pathology and the disorganized neurofilaments can induce selective neuronal degeneration and death [24].

It has been suggested that reactive glial cells could help protect retinal ganglion cells, as they can be a source of neurotrophic factors [25]. On the contrary, reactive glial cells can exacerbate neuronal damage and may become one of the etiologies of glaucoma through the release of cytokines, reactive oxygen species, and functional disorders of the glutamate uptake in Müller cells [26,27]. This could negatively influence ganglion cells, which could lose their normal functional support [28,29]. In this regard, the colocalization of caspase 3 and GFAP in astrocytes and Müller glia in glaucomatous retina has indicated that these cells may be involved in the apoptosis process, in which the increase of nitric oxide (NO) and tumor-necrosis factor (TNF-α) produced by glial cells would lead to the death of retinal ganglion cells exposed to stressful conditions [30,31].

Altered crosstalk between RGCs and microglia, astrocytes or oligodendrocytes has been proposed as an early factor in the pathophysiology of glaucoma [20]. The lack of agreement concerning the role played by the glia in ganglion cells has raised the need for research on both the location and the discrimination of responses which take place simultaneously in the RGCs and glia [8].

The present study analyzes a mouse model of ocular hypertension (OHT), both in the eye with laser-induced OHT (OHT-eyes) and in the contralateral eye. The aim was to determine i) concurrent responses of macroglial and retinal ganglion cells, using specific antibodies as markers against cytoskeletal proteins from both cells: GFAP (a major constituent of the astrocyte cytoskeleton [32] and NF-200kD (a major constituent of the neuronal cytoskeleton, which after axonal injury has an abnormal distribution in the soma and dendrites of the RGCs [33]; and ii) whether there is an inflammatory reaction to OHT by using anti-Iba 1 (a retinal microglial-specific calcium-binding adaptor protein) [34,35] and an antibody against the class II major histocompatiblity complex (anti-MHC-II) (a marker for active antigen-presenting cells) [36] and then compare them with retinas from naïve eyes.

## Methods

### Animals and anesthetics

Experiments were performed on adult male albino Swiss mice (40 to 45 g) obtained from the breeding colony of the University of Murcia (Murcia, Spain). The animals were housed in temperature- and light-controlled rooms with a 12-hour light/dark cycle and *ad libitum* access to food and water. Light intensity within the cages ranged from 9 to 24 luxes. Animal manipulation followed institutional guidelines, European Union regulations for the use of animals in research, and the ARVO (Association for Research in Vision and Ophthalmology) statement for the use of animals in ophthalmic and vision research. All surgical procedures were performed under general anesthesia induced with an intraperitoneal (i.p.) injection of a mixture of Ketamine (75 mg/kg, Ketolar®, Parke-Davies, S.L., Barcelona, Spain) and Xylazine (10 mg/kg, Rompún®, Bayer, S.A., Barcelona, Spain). During recovery from anesthesia, mice were placed in their cages and an ointment containing tobramycin (Tobrex®; Alcon S.A., Barcelona, Spain) was applied on the cornea to prevent corneal desiccation and infection. Additional measures were taken to minimize discomfort and pain after surgery. The animals were killed with an i.p. overdose of pentobarbital (Dolethal Vetoquinol®, Especialidades Veterinarias, S.A., Alcobendas, Madrid, Spain).

### Experimental groups

Two groups of mice were considered for study: an age-matched control (naïve, n = 9) and a lasered group (n = 9). This latter were processed two weeks after lasering.

### Induction of ocular hypertension and IOP measurements

To induce OHT, the left eyes of anesthetized mice were treated in a single session with a series of diode laser (Viridis Ophthalmic Photocoagulator-532 nm, Quantel Medical, Clermont-Ferrand, France) burns. The laser beam was directly delivered without any lenses, aimed at the limbal and episcleral veins. The spot size, duration, and power were 50 to 100 μm, 0.5 seconds and 0.3 W, respectively. Each eye received between 55 to 76 burns.

The intraocular pressure (IOP) of the mice was measured under deep anesthesia in both eyes with a rebound tonometer (Tono-Lab, Tiolat, OY, Helsinki, Finland) [30,37] prior to and 24 to 48 hours and one week after laser treatment for the lasered group and before being killed for the naïve. At each time point, 36 consecutive readings were made for each eye and averaged. To avoid fluctuations of the IOP due to the circadian rhythm in albino Swiss mice [38] or due to the elevation of the IOP itself [39], we tested the IOP consistently around the same time, preferentially in the morning and directly after deep anesthesia in all animals (lasered group and naïve). Moreover, because general anesthesia lowers the IOP in the mouse, we measured the IOP of the treated eye (OHT-eye) as well as the contralateral intact fellow eye in all the experiments.

### Immunohistochemistry

The mice were deeply anesthetized, perfused transcardially through the ascending aorta first with saline and then with 4% paraformaldehyde in 0.1 M phosphate buffer (PB) (pH 7.4). The orientation of each eye was carefully maintained with a suture placed on the superior pole immediately after deep anesthesia and before perfusion fixation. Moreover, upon dissection of the eye, the insertion of the rectus muscle and the nasal caruncle were used as additional landmarks [40]. The eyes were post-fixed for two hours in the same fixative and kept in sterile 0.1 M PB.

The retinas from both groups were dissected and processed as retinal whole-mounts [41]. Of the nine mice included in the lasered group, six were used to quantify the effect of OHT on astrocytes and RGCs while three were used to analyze whether there was an inflammatory response to OHT. The retinas of the mice were double immunostained as described elsewhere [42] with anti-GFAP plus anti-NF-200 (which recognizes both phosphorylated and dephosphorylated forms of the 200-kD neurofilaments) in order to study the effect of OHT on retinal macroglia and RGC, respectively. The working dilutions were 1/80 for rabbit anti-neurofilament 200 (Sigma-Aldrich, Tres Cantos, Madrid, Spain) and 1/150 for mouse anti-GFAP (GFAP clone GA-5) (Sigma-Aldrich, Tres Cantos, Madrid, Spain). Binding sites of the primary antibodies were visualized after two days of incubation with the corresponding secondary antibodies: the immunoglobulin fraction of goat anti-mouse antibody conjugated to fluorescein isothiocyanate (FITC) (Sigma, St. Louis, Missouri, USA) diluted 1/100 and goat anti-rabbit antibody conjugated to Texas-red (Vector, Burlingame, CA, USA) diluted 1/50. Vectashield mounting medium for fluorescence with nuclear counterstaining (4',6-diamidino-2-phenylindole, DAPI) (Vector, Burlingame, CA, USA) was used to distinguish one astrocyte clearly from another for counting purposes.

To determine whether there was an inflammatory reaction to OHT, we triple immunostained the retinal whole-mounts with the following primary antibodies: anti-mouse MHC class II (I-A/I-E) (eBioscience, San Diego, CA, USA) in a 1/100 dilution, rabbit anti Iba 1 (Wako, Osaka, Japan) in a 1/500 dilution and chicken anti-GFAP (Millipore, Massachusetts, MA, USA) in a 1/100 dilution. Binding sites of the primary antibodies were visualized with the corresponding secondary antibodies: goat anti-mouse Alexa Fluor 488 (Invitrogen, Paisley, UK) diluted 1/150, donkey anti-rabbit Alexa Fluor 594 (Invitrogen) diluted 1/800 and DyLight 405-conjugated donkey anti-chicken (Jackson ImmunoResearch, West Grove, PA, USA) diluted 1/150. Negative controls included replacement of primary and secondary antibodies by normal serum from those species in which the primary antibodies were raised [42].

### Retinal analysis

#### GFAP-labeled retinal area (GFAP-RA). Intensity of GFAP immunoreaction (GFAP-IR). Astrocyte and NF-200+RGC counting

Mice retinal whole-mounts were examined and photographed under a confocal microscope (Leika TCS SP2 AOBS) and a fluorescence microscope (Zeiss, Axioplan 2 Imaging Microscope) equipped with appropriate filters for fluorescence emission spectra of FITC (Filter set 10, Zeiss) and Texas-red (Filter set 15, Zeiss). Fluorescence microphotographs were taken with the same exposure time (700 ms). Retinal astrocytes and RGCs were quantified by a masking procedure.

To determine the effect of OHT, we quantified astrocytes and NF-200+RGC somas in the retinal whole-mount as follows: In an initial step, NF-200+RGC somas were counted and measured in the retinal whole-mount. Each entire retinal whole-mount was analyzed using the motorized stage of the microscope to scan the whole preparation along the x-y axis. Thus, all subsequent fields analyzed were contiguous and were examined systematically to ensure that no portion of the retinal whole-mount would be omitted or duplicated. Additionally, so as not to undersample labeled NF-200+RGC somas lying outside the immediate focal plane, we analyzed the whole preparation along the z axis. The NF-200+RGC somas were counted and their size was calculated with the manual counting tool and the measuring tool, respectively, included in the Metamorph Imaging System version 4.5 computer program (© Universal Imaging Corps) in association with an Axioplan 2 Imaging Microscope (Zeiss). The diameter used to estimate NF-200+RGC soma size was the longest distance between opposing cell boundaries when passing through the center of the cell. These procedures were made at 20x, giving up an area of 0.19 mm^2^ per fields analyzed.

**Figure 1:**
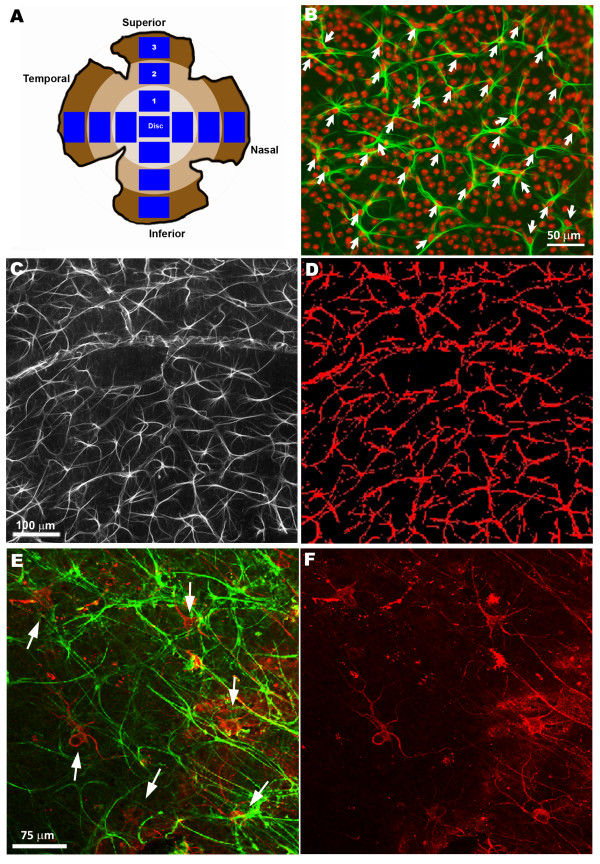
**Retinal whole-mount. GFAP-labeled retinal area (GFAP-RA) and NF-200+RGC counting. A**: division of the retina in concentric zones for study and areas of retina selected from each zone; **B**: photomicrograph illustrating the astrocyte-counting methodology, GFAP+ astrocytes (in green) and DAPI, a nuclear marker (in red); **C**: photomicrograph of one of the areas of the retina selected for GFAP-RA quantification; **D**: same area shown in C processed with the threshold tool included in the Metamorph Imaging System. In red, GFAP-labeled retina included in the measurements and processing; **E**: double immunostaining photographs used to correlate the effect of OHT in both GFAP+ astrocytes (in green) and NF-200+RGCs (in red); **F**: same area shown in E used for manual counting of NF-200+RGCs. Fluorescence microscopy (B,C,D); Confocal microscopy (E,F). GFAP, glial fibrillary acidic protein; NF-200, 200 kD neurofilament; OHT, ocular hypertension; RGCs, retinal ganglion cells.

In the second step, to evaluate the effect of OHT in astrocytes, equivalent areas of the retina were consistently selected for each retinal whole-mount, which included the optic disc, superior, inferior, nasal, and temporal zones of the retina (13 areas in total for each retina; Figure 1A). Photographs of these areas were taken at 10x, providing an area of 0.78 mm^2^. Astrocytes in mice retina are distributed in such a way that each GFAP-labeled astrocyte can be easily distinguished from others, allowing cell counting. Astrocytes were counted in each selected photograph by using the manual counting tool of the Metamorph Imaging System (Figure 1B).

In addition, to analyze the effect of OHT in GFAP, we used the same selected photographs to determine the GFAP-labeled retinal area (GFAP-RA). For this purpose, we used a computer-assisted morphometric analysis to quantify the retinal area stained with GFAP. The images were thus processed with the Threshold Tool of the Metamorph Imaging System. Thresholding defines a range of gray-scale values found on the pixels of objects of interest, differentiating them from other parts of the image based on the images’ gray scale. Areas of the image that were marked with the red threshold overlay (GFAP+ astrocytes and GFAP+ end-foot of the Müller cells; Figure 1C and D) as a visual indicator of the thresholded areas were included in the measurement and processing [43]. Individual images were taken with a digital high-resolution camera (CoolSNAP Photometrics USA) and further processed when required using Adobe Photoshop® CS3 Extended 10.0 (Adobe Systems, Inc., San Jose, CA, USA). Additionally, to correlate the effect of OHT in both astrocytes and RGCs, NF-200+RGC somas were manually counted in the 13 retinal areas used for the astrocyte study (Figure 1E and F).

**Figure 2:**
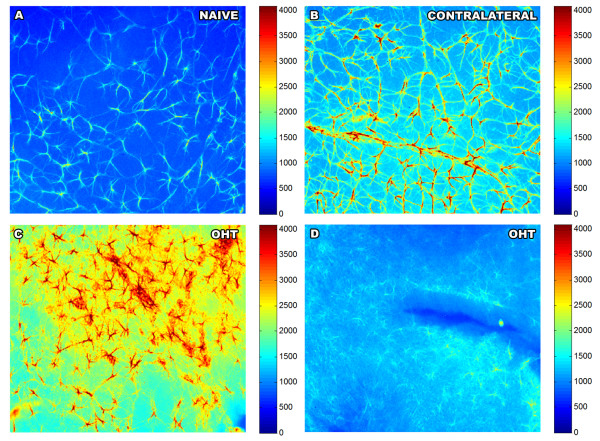
**Intensity of GFAP immunoreaction. Pseudocolor-intensity maps drawn with MATLAB program.** All maps had the same preset color scale that assigned a color to each intensity value, identifying different intensity levels of the GFAP-IR (Müller cells plus astrocytes). Cool colors represent lower intensity levels while warm colors represent higher intensity levels. Although no differences in mean intensity of GFAP-IR were found between naïve (**A**) and contralateral (**B**) eyes, differences were found when the comparison was made by the percentage of the retinal area occupied by GFAP+ cells with higher intensity levels. **C**-**D** microphotographs were taken from the same retina. They illustrate that OHT-eyes had retinal areas with high (C) and low (D) intensity of GFAP-IR, the later having mainly astrocytes in which only primary processes were detected. GFAP-IR, glial fibrillary acidic protein immunoreaction; OHT, ocular hypertension.

Finally, photographs taken at 10x were used to determine the intensity of the GFAP-IR. For this, we used MATLAB (© MathWorks, Inc) and the Metamorph Imaging System. MATLAB is a high-level technical computing language that can be used for image processing. Some tools of the program allowed us to create absolute pseudocolor intensity maps based in a gray scale ranging from zero to 4,095 (12 bits images). All maps had the same preset color scale that assigned a color to each intensity value (Figure 2), allowing us to identify different intensity levels of the GFAP-IR (Müller cells plus astrocytes) among groups of study. In addition, MATLAB was used to quantify the mean intensity of the GFAP-IR and the percentage of the retinal area occupied by GFAP+ cells with higher intensity levels.

The GFAP intensity of individual astrocytes in OHT-eyes was quantified in each selected photograph by using the pixel-value information associated with the manual counting tool of the Metamorph Imaging System.

#### Analysis of Iba-1 and MHC-II expression

Samples processed with MHC-II and Iba-1 antibodies were analyzed and photographed with the ApoTome device (Carl Zeiss, Germany) coupled to a fluorescence microscope (Zeiss, Axioplan 2 Imagin Microscope) equipped with appropriate filters for fluorescence-emission spectra of Alexa fluor 488 (Filter set 10, Zeiss), Alexa fluor 594 (Filter set 64, Zeiss) and DyLight 405 (Filter set 49, Zeiss). The ApoTome uses the ‘structured-illumination’ method that enables conventional microscopy to create optical sections through the specimen and thereby improve the contrast and resolution along the optical axis.

### Statistical analysis

The selected areas of the retina that were taken at 10x were used to quantify the number of astrocytes, GFAP-RA, and the number of RGCs. These were grouped in two different ways for analysis: as areas of the retina (disc, superior, inferior, nasal, and temporal, giving rise to 13 areas per retina) and concentric zones of the retina (disc, central, intermediate and periphery).

Data for the statistical analysis were introduced and processed in a SPSS 19.0 (comprehensive statistical software; SPSS Inc^©^). Data are shown as mean ± SD. Statistical analyses were performed with the analysis of variance (ANOVA) and Bonferroni test to identify differences among: i) IOP values of the OHT-eyes, the contralateral and naïve eyes; ii) the GFAP-labeled retinal area (GFAP-RA); iii) mean intensity of GFAP-staining; iv) the percentage of the retinal area occupied by GFAP+ cells with higher intensity levels (above 3,000 in the gray scale); and v) astrocyte number. A *T* test was used to compare: i) the GFAP-RA between the contralateral and the naïve eyes; ii) the number of astrocytes in which primary and secondary processes could be identified between OHT-eyes and naïve; iii) the percentage of the retinal area occupied by GFAP+ cells with higher intensity levels (above 3,000 in the gray scale) between the contralateral and the naïve eyes; and iv) the intensity of GFAP-IR between astrocytes in OHT-eyes in which primary and secondary processes could be identified and those in which only primary processes could be identified. Pearson’s correlation was used to assess the possible relation between IOP and: i) the GFAP-RA of eyes from lasered eyes; ii) the number of astrocytes in which only primary processes could be identified in OHT-eyes; and iii) the number of NF-200+RGCs in OHT-eyes. The same correlation was used to determine the possible relation between NF-200+RGCs and: i) the GFAP-RA in OHT-eyes; and ii) the number of astrocytes in which only primary processes could be identified in OHT-eyes.

## Results

### Laser-induced ocular hypertension

The IOP values of OHT-eyes (29.6 ± 4.4 mmHg) significantly differed from naïve values (16.2 ± 3.1 mmHg; *P* < 0.001, ANOVA with Bonferroni) and contralateral eyes (15.4 ± 1.6 mmHg; *P* < 0.001, ANOVA with Bonferroni). No significant differences were found between contralateral and naïve eyes.

### Effects of 15 days of OHT in retinal macroglia (GFAP expression)

**Figure 3:**
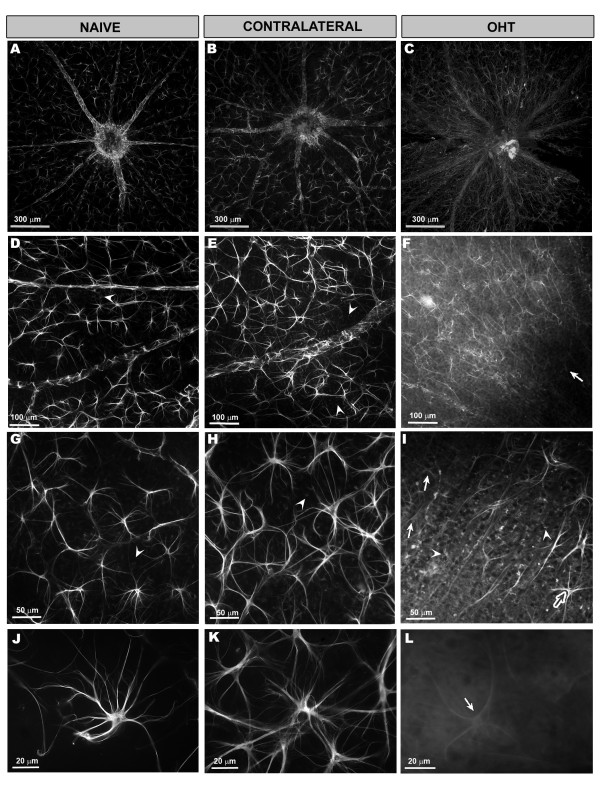
**GFAP immunostaining of equivalent areas of the retinal whole-mounts.** Astrocyte morphology and GFAP-IR of macroglial cells in naïve and in contralateral and OHT-eyes after 15 days of laser-induced OHT. **A-C**: overview of the retinal astrocytes around the optic disc in naïve (A), contralateral (B), and OHT-eyes (C). **D-L**: images correspond to zone 1 of study. In naïve eyes (A, D, G, J), astrocytes formed a homogeneous plexus on the nerve-fiber-RGC layer of cells regularly distributed throughout the retina. This plexus was constituted by stellate cells that could easily be distinguished from each other (D, G). Astrocytes had a rounded body from which numerous primary and secondary processes extended (J) and Müller cells (arrowhead) exhibited punctate GFAP+ structures between the astrocytes (D, G). In contralateral eyes (B, E, H, K), astrocytes were more robust (K) than in naïve eyes (J) and formed a honeycomb network (E, H, K). GFAP+ Müller cells were observed in some retinal areas (arrowhead) in (E, H). Astrocyte morphology in OHT-eyes (C, F, I, L) was not uniform, with astrocytes in which primary and secondary processes could be observed (empty arrow) in (I) and astrocytes in which only primary processes could be observed (arrow) in (I and L). GFAP+ Müller cells (C, F, I) were visible throughout the retina (arrowhead) in (I). Confocal microscopy (A-I); Fluorescence microscopy (J-L). GFAP-IR, glial fibrillary acidic protein immunoreaction; OHT, ocular hypertension; RGC, retinal ganglion cells.

Morphological description

i. *Age-matched control (naïve)*: In naïve mice the astrocytes formed a homogeneous plexus on the nerve-fiber-RGC layer of GFAP+ cells regularly distributed throughout the retina from the disc (Figure 3A) to the periphery. This plexus was constituted by stellate cells that could be easily distinguished from each other, allowing the possibility of manually counting individual cells (Figure 3D, G and J). GFAP+ astrocytes had a rounded body from which numerous primary and secondary processes extended (Figure 3J). In some retinal areas, the Müller cells were GFAP+ and appeared as punctate structures between the astrocytes and their radiating processes (Figure 3D).

ii. *Contralateral eyes:* astrocytes had a rounded cell body from which numerous primary and secondary processes extended. Unlike in naïve eyes, astrocytes were more robust (Figure 3K) and formed a honeycomb network (Figure 3B, E and H). Similar to naïve eyes, GFAP+ Müller cells were observed in some retinal areas (Figure 3E and H).

iii. *Treated eyes (OHT-eyes):* we found astrocytes in which primary and secondary processes could be observed at 10x (Figure 3I) and others in which only primary processes could be recognized at this magnification (Figure 3I and L). GFAP+ Müller cells were visible throughout the retina (Figure 3C, F and I).

**Figure 4:**
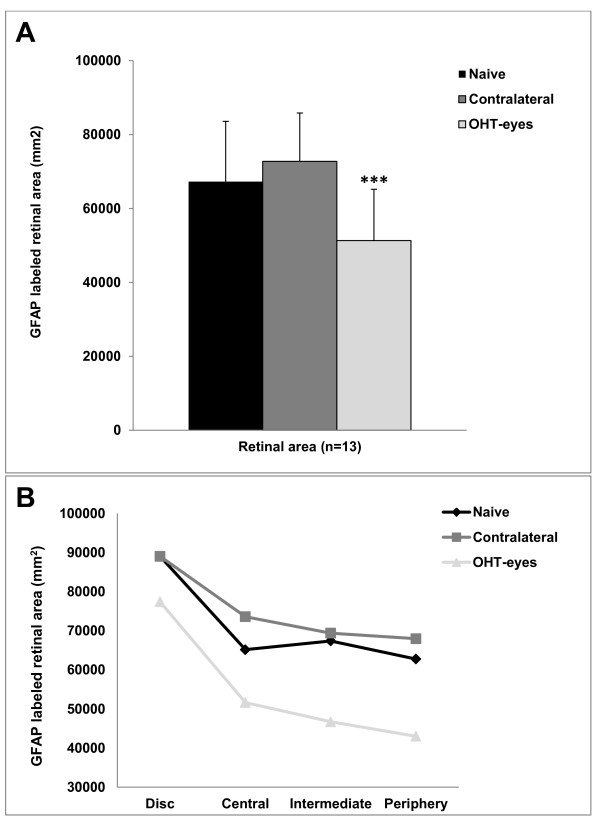
**GFAP-labeled retinal area (GFAP-RA) after 15 days of laser-induced OHT.** Comparison among areas and concentric zones of the retina analyzed in the three study groups. The GFAP-RA of the OHT-eyes underwent a statistically significant reduction in comparison with naïve and contralateral eyes. This finding was observed when the analysis was made both by retinal areas (13 target areas per retina) (**A**) and by concentric zones of the retina (disc, central, intermediate, and periphery) (**B**). Each bar represents the mean ± SD of GFAP-RA. ***P < 0.001 versus naïve and contralateral retinas. ANOVA with Bonferroni test. ANOVA, analysis of variance; GFAP, glial fibrillary acidic protein; OHT, ocular hypertension.

Astrocyte number and GFAP-labeled retinal area (GFAP-RA)

i. *Astrocyte number:* The astrocyte number did not differ significantly among the eyes analyzed (215 ± 14; 202 ± 19; 208 ± 30 for naïve, contralateral, and OHT-eyes, respectively). However, in OHT-eyes the number of astrocytes in which primary and secondary processes could be observed at 10x (129 ± 20) were decreased in comparison with naïve eyes (215 ± 14) (*P* < 0.01, unpaired *T* test). In the six experimental retinas with OHT included in the quantitative study, the mean percentage of astrocytes in which only primary processes could be detected at 10x was 37.8% (78.6 of 208).

ii. *GFAP-RA:* The GFAP-RA in OHT-eyes (51,869 ± 5,461) was reduced in comparison with contralateral eyes (*P* < 0.001) and naïve eyes (*P* < 0.001) (ANOVA with Bonferroni test) (Figure 3A-I). This difference was observed when the analysis was made both by areas of the retina (n = 13) (Figure 4A) and by concentric zones (n = 4) (Figure 4B). In contrast to OHT-eyes, the contralateral eyes had significantly more GFAP-RA (72,783 ± 13,061) than naïve (67,283 ± 16,318) (*P* < 0.05, unpaired *T* test) (Figure 3D, E, G, H, J, K).

The analysis of GFAP-RA by zones in each group showed that the GFAP-RA differed among zones in the three groups analyzed (naïve, contralateral, and OHT-eyes: *P* < 0.003; *P* < 0.002 and *P* < 0.000, respectively. ANOVA). The Bonferroni test showed that the optic disc was the only retinal zone that contained significantly more GFAP-RA than the others (*P* < 0.01, *P* < 0.05, *P* < 0.001 for naïve, contralateral and OHT-eyes, respectively). Both in OHT-eyes and in contralateral eyes the GFAP-RA from the concentric zones chosen for study tended to decrease from the disc to the periphery (Figure 4B).

#### Intensity of GFAP-IR

The comparison of the mean intensity of GFAP-IR revealed that the three groups of study eyes differed from each other (*P* < 0.01; ANOVA). The Bonferroni test showed that in OHT-eyes (2,019 ± 392) it was significantly higher than in naïve (1,330 ± 162) (*P* < 0.01). However, when we considered only those retinal areas with the highest intensities of GFAP-IR, we found that in contralateral eyes the mean percentage of the retinal area occupied by GFAP + cells with intensities above 3,000 was higher than in naïve eyes (2.0 ± 1.8 and 0.2 ± 0.3, respectively; *P* = 0.05; unpaired *T* test) but it did not differ from values for OHT-eyes (5.6 ± 4.3) (Figure 2).

The analysis of OHT-eyes revealed that the intensity of GFAP-IR in astrocytes in which primary and secondary processes could be observed at 10x (3,021 ± 534) (Figure 2C) was significantly higher than those in which only primary processes could be recognized at the same magnification (2,073 ± 497) (*P* < 0.001; paired *T* test; Figure 2D).

### **Effects of 15 days of OHT in the retinal ganglion cells (NF-200 expression)**

**Figure 5:**
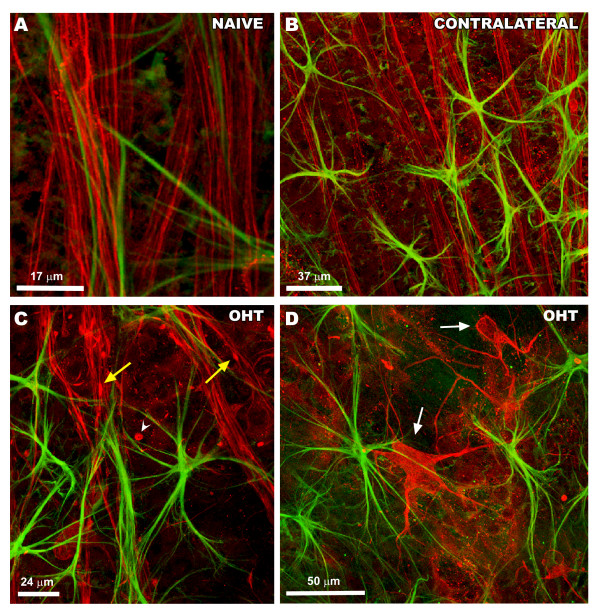
**Retinal whole-mount. Double immunostaining for NF-200 and GFAP after 15 days of laser-induced OHT.** Axon and RGCs (in red). Macroglia (in green). **A-C**: Images correspond to area 1 of study. **D**: image corresponds to area 3 of the study. A-B: NF-200 labelling of RGC axons was rectilinear and uniform in naïve (A) and contralateral eyes (B). NF-200+RGC somas were infrequently observed; C-D: beads on a string-like immunostaining (arrowhead) and small varicosities (yellow arrow) in NF-200+ axons in OHT-eyes. Abnormal staining of cell bodies and primary dendrites of RGCs (white arrow) were more frequently found in OHT-eyes (D) than in naïve and contralateral eyes. Confocal microscopy. GFAP, glial fibrillary acidic protein; OHT, ocular hypertension; RGCs, retinal ganglion cells.

Morphological description

i. *Naïve and contralateral eyes:* RGC axons were uniformly labeled with anti-NF-200, and their morphology was rectilinear. NF-200+RGC staining was rarely observed in the somas or dendrites of RGCs (Figure 5A and B).

ii. *OHT-eyes:* We observed abnormal NF-200 accumulation both in RGC axons (beads on a string and small varicosities) as well as in the cell bodies and primary dendrites of some RGCs (Figure 5C and D). NF-200 labeling within the cell soma and primary dendrites varied from a faint but clear staining to an intense labeling (Figure 5D).

#### RGC counting and size

**Figure 6:**
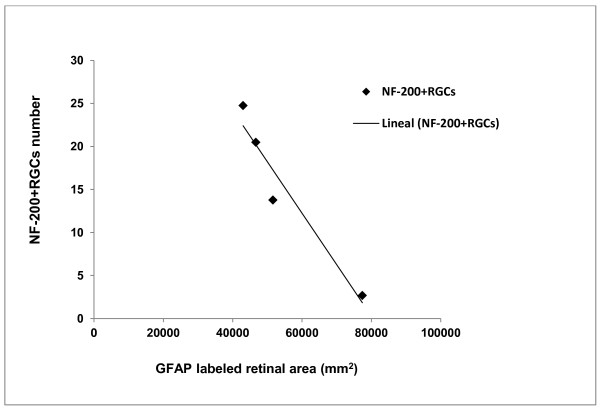
**Correlation of GFAP-labeled retinal area (GFAP-RA) versus NF-200+RGC number in OHT-eyes after 15 days of laser-induced OHT.** There was a trend for the GFAP-RA to decrease and for the NF-200+RGC number to increase from the disc to the periphery in OHT-eyes. Data regarding GFAP-RA are the same as in Figure 3. OHT, ocular hypertension; RGCs, retinal ganglion cells.

In the six naïve retinas used for the quantitative study, the number of NF-200+RGCs in each was 0, 0, 1, 2, 2, 3, respectively, with a mean of 1.3 ± 1.2. Only one of the six contralateral retinas quantified had one NF-200+RGC. The retina of one of the six eyes with OHT was not suitable for NF-200+RGC counting in the whole retina. In the remaining five OHT-eyes, the number of NF-200 + RGCs found was 520, 611, 259, 509, and 616, with a mean of 503 ± 145.16. There was a trend (r = −0.47, *P* < 0.01 Pearson’s correlation) for the GFAP-RA to decrease and for the NF-200+RGC number to increase from the center to the periphery (Figure 6).

NF-200+RGC somas size in OHT-eyes ranged from 11 μm to 45 μm. Cells ranging from 17 to 24 μm accounted for 58.2% of NF-200+RGCs.

### Effects of 15 days of OHT in the Iba-1 and MHC-II expression

#### Age-matched control (naïve)

**Figure 7:**
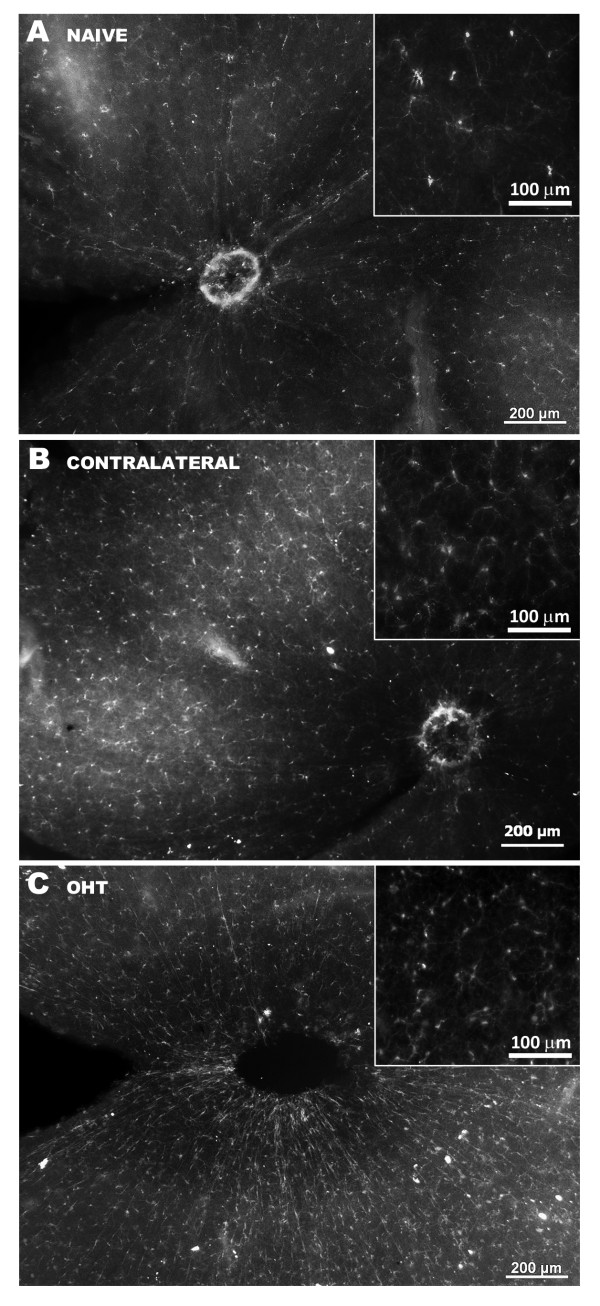
**Iba-1+ cells in the retinal whole-mounts after 15 days of laser-induced OHT.** The density of Iba-1+ network was increased in contralateral (**B**) and OHT-eyes (**C**) with respect to naïve (**A**). Higher magnification of equivalent fields in the retinas correspond to area 2 (insets). A-C: 5x; Insets: 10x. Fluorescence microscopy. Iba-1, ionized calcium binding adaptor molecule 1; OHT, ocular hypertension.

**Figure 8:**
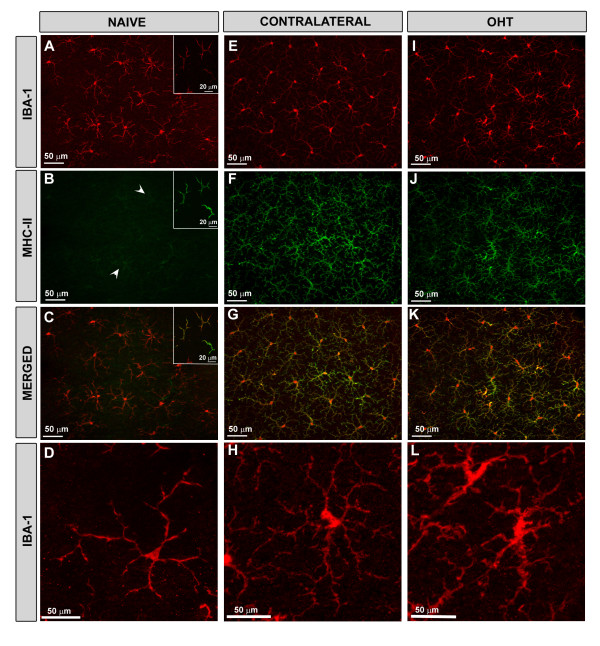
**MHC-II expression of Iba-1+ cells in retinal whole-mounts after 15 days of laser-induced OHT.** Double immunofluorescence staining for Iba-1 (in red) and MHC-II (in green). **A-C**: in naïve retinas, a constitutive weak expression of MHC-II (arrowheads) was observed in some Iba-1+ cells throughout the retina (B). However, some Iba-1+ cells located in the juxtapapillary area and in the far periphery (A, inset) had a strong constitutive expression of MHC-II (B, inset). **E-H**: in contralateral eyes, Iba-1+ microglial cells of the retina had morphological changes (H) and showed a stronger expression of MHC-II immunoreaction throughout the retina (F,G) in comparison with naïve (B-D). **I-L**: Iba-1+ cell in OHT-eyes had a similar up-regulation of MHC-II (J-K) as did contralateral eyes (F-G); however, the cell bodies were larger and the processes thicker and more retracted (L) than in the contralateral (H) retinas. Fluorescence microscopy and image acquisition using the ApoTome. Iba-1, ionized calcium binding adaptor molecule 1; MHC, major histocompatibility complex; OHT, ocular hypertension.

Retinal microglia from naïve eyes stained with anti-Iba-1 exhibited morphological features typical for this cell type (that is, several thin primary processes emanating from small cell bodies with a ramification at many branching points). Microglia cells were distributed in a parallel mosaic of tiled cells that built networks throughout the entire retina without overlap between their processes (Figures 7A, 8A).

**Figure 9:**
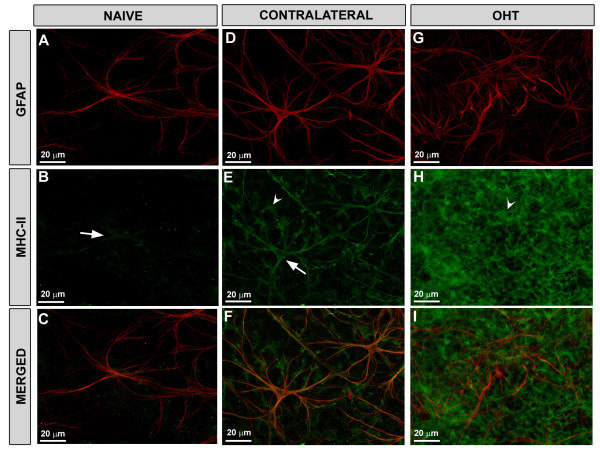
**Retinal whole-mount. Double immunostaining for GFAP (red) and MHC-II (in green) after 15 days of laser-induced OHT**. **A-C**: naïve eyes; **D-F**: contralateral eyes; **G-I:** OHT-eyes. In contralateral eyes MHC-II immunoreaction of astrocytes (arrow) and Müller cells (arrowhead) in **(E)** was increased with respect to naïve eyes (arrow) in (**B**). In OHT-eyes, MHC-II immunoreaction of Müller cells (arrowhead) in **(H)** was notably upregulated in comparison with contralateral (**E**). Fluorescence microscopy and image acquisition using the ApoTome. GFAP, glial fibrillary acidic protein; MHC, major histocompatibility complex; OHT, ocular hypertension.

Weak constitutive MHC-II expression was found in some microglial cells (Figure 8B and C) and only rarely in astrocytes (Figure 9B and C) in the naïve retina. Only a small subpopulation of Iba-1+ cells that exhibited dendritiform morphology and that was located in the juxtapapillary area as well as in the marginal region of the retina, had strong MHC-II immunoreaction (Figure 8B inset and 8C inset). We detected no MHC-II immunostaining in Müller cells.

#### Contralateral eyes

**Figure 10:**
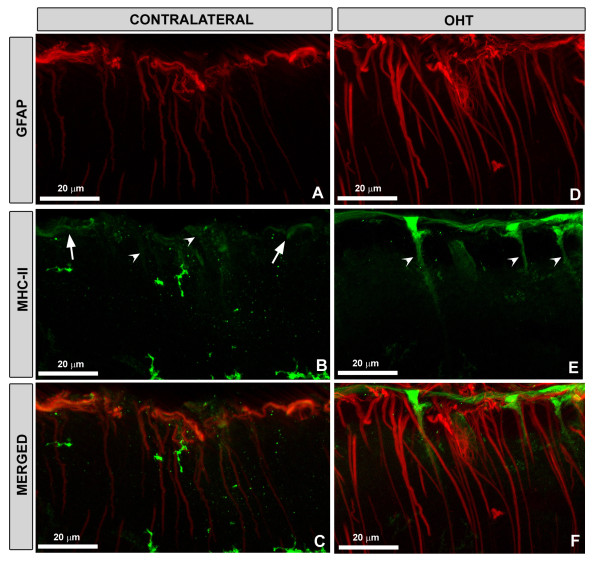
**Retinal whole-mount. Double immunostaining for GFAP (in red) and MHC-II (in green) after 15 days of laser-induced OHT.** The pressure exerted by the cover glass on the retinal whole-mount, produced a retinal-like section effect in some retinal borders. **A-C:** in contralateral eyes (A), the GFAP immunoreaction (IR) of Müller cells was weaker than in OHT-eyes (D). MHC-II expression was greater in astrocytes (arrow) than in Müller cells (arrowhead) in (B); **D-F**: in OHT-eyes, MHC-II was upregulated in Müller cells (arrowhead) in (E), the intensity of the staining being greater than in contralateral eyes (arrowhead) in (B). GFAP-IR was absent or faint in those Müller cells exhibiting MHC-II immunostaining (D-F). Both in contralateral (B) and OHT-eyes (E), the retinal-like section effect enabled us to determine that MHC-II upregulation in Müller cells was preferentially located in the end-foot of the cells. Fluorescence microscopy and image acquisition using the ApoTome. GFAP, glial fibrillary acidic protein; MHC, major histocompatibility complex; OHT, ocular hypertension.

At low magnification (5x), Iba-1+ cells in the contralateral (Figure 7B) retinas formed a denser network than in naïve retinas (Figure 7A). Their morphology differed with regard to naïve in that the somas were larger and the primary and secondary processes were thicker and more branched, with fine, finger-like extensions from the major branches (Figure 8E and H). In comparison to the naïve retina, MHC-II expression in macroglia and microglia of contralateral retinas was upregulated. Overall, Iba-1+ cells were labeled strongly with MHC-II throughout the contralateral retina (Figure 8F and G). MHC-II immunostaining of astrocytes was visible throughout the retina (Figures 9E, F, 10B and C). By contrast, MHC-II immunoreaction of Müller cells was weaker than in astrocytes (Figure 10B and C) and was restricted to some retinal areas (Figure 9E and F).

#### Treated eyes (OHT-eyes)

At low magnification (5x) the retinal network formed by microglial cells was denser (Figure 7C) than in the contralateral (Figure 7B) and naïve eyes (Figure 7A). These cells had morphological signs of activation, exhibiting larger cell bodies and thicker and retracted processes (Figure 8I and L) than in contralateral (Figure 8E and H) and in naïve eyes (Figure 8A and D).

Most Iba-1+ microglia showed high MHC-II immunoreaction (Figure 8J and K) similar to contralateral (Figure 8F and G). No MHC-II astrocytes were found. Müller cells showed higher immunoreaction for MHC-II (Figure 9H and I) than contralateral (Figure 9E and F). MHC-II expression was detected in several groups of Müller cells throughout the retina, preferentially located in the end-foot of the cells (Figure 10E and F). Notably, GFAP-IR was absent or faint in Müller cells exhibiting MHC-II immunostaining (Figure 10D-F).

## Discussion

Experimental rodent models have been used to study glaucomatous neuropathy because they are inexpensive and easier to handle than other animal models (dog and rabbit). In comparison with the rat, the mouse has a major advantage, which is the possibility of being genetically manipulated [44]. In addition, although GFAP+ astrocytes were quantified in rats [45,46], another advantage is that mouse astrocytes are farther apart from each other, allowing us to identify individual cells and count them one by one, more accurately than in rats [43].

Findings in mouse retinal glial cells reported here correspond to changes observed 15 days after lasering the treated eye. In the model of laser-induced OHT used in the present work, a substantial increase of the IOP was evident 24 hours after lasering which continued for four days and then gradually returned to the basal value after the fifth day, so that by one week after lasering, the IOP values in the treated animals were comparable for both eyes [40]. The experimental conditions of the present study constitute a model for human glaucomatous optic neuropathy and thus, can be used to investigate OHT-induced changes undergone by retinal macroglia, microglia, and ganglion cells.

The main reason to use whole-mounted preparations of the retina was to quantify the population of astrocytes and NF-200+RGCs in the whole mouse retina and also to analyze the behavior of glial cells and NF-200+RGCs in different retinal zones. In addition, whole-mounted preparations allow the differentiation of astrocytes from Müller glial cell end-feet which otherwise are not readily distinguishable in a sectional profile [47,48], allowing astrocyte quantification.

The intermediate filament protein GFAP of astrocytes is considered an early marker for retinal injury and is commonly used as an index of gliosis-hypertrophy [21,49,50]. Two relevant morphological alterations in gliotic Müller cells are hypertrophy and the expression of the filament protein GFAP [51].

It has been reported that experimental diabetes in rats induces a differential GFAP expression pattern in the macroglial cells of the retina, reduces GFAP-IR in astrocytes, and increases GFAP-IR in Müller cells [47]. Such opposite reactions in astrocytes and Müller cells in terms of GFAP-IR has been reported in OHT-eyes of two models of experimental glaucoma in rats [43,45]. In the present study, a similar behavior took place in the macroglial of OHT-eyes in mice.

Numerous *in vitro* and *in vivo* studies have shown GFAP to be essential for several astrocyte functions such as proliferation, differentiation, extension of processes, vesicle trafficking, astrocyte-neuron interaction [52], astrogliosis [53] and protection from cerebral ischemia [52].

In comparison to the naïve group, in some astrocytes of OHT-eyes the secondary processes could not be identified at 10x magnification. This difference could have contributed to the decreased GFAP-RA found in OHT-retinas. In addition, the intensity of the GFAP-IR in OHT-eyes was significantly higher than in naïve eyes. Whether or not astrocyte differences in OHT-eyes reflect a functional change, that is, preparation for migration or a switch from neuroprotective functions to immunogenic functions or astrocyte damage, is unknown. Astrocyte changes in OHT-eyes could impair the neurosupportive role of astrocytes [54] and participate in the death of RGCs reported in a recent parallel study using a comparable methodology to induce OHT [40].

It bears noting that the astrocyte number was similar in the three groups studied (OHT-eyes, contralateral, and naïve eyes). This leads us to postulate that in the retina of OHT-eyes a reactive, non-proliferative gliotic response takes place, similar to that reported in mouse [34,55] and rat [43,55]. It has been suggested that a gliotic non-proliferative response is the consequence of slow degeneration, while rapid degeneration leads to a proliferative gliosis [34,55,56]. However, it should be stressed that, although the number of astrocytes in OHT eyes did not differ significantly from naïve, 37.8% of the astrocytes exhibited morphological changes that could account for the decrease of GFAP-RA in eyes with OHT.

NF-200 is a component of the neuronal cytoskeleton. Under normal conditions, anti-NF-200 labels axons of the RGCs but rarely labels RGC somas [57]. Both staining patterns were observed in our naïve group. It is known that an elevated IOP has been associated with the disruption of the axonal transport [58] in different animal models [2,59-62]. A factor deeply involved in axonal transport is phosphorylation of the heavy neurofilament subunit (NF-H) [63-65]. In a monkey model of chronic ocular hypertension [66], most NF-Hs in RGC axons in the glaucomatous eyes were significantly dephosphorylated by high IOP, which may be a sign of damaged axonal transport. In the present study, the number of NF-200+RGCs was significantly increased in OHT-eyes in comparison to naïve and contralateral eyes. These RGCs that had accumulated NF-200 in their cell bodies, proximal axon, and primary dendrites most probably represented functionally impaired RGCs as a consequence of the disruption of the axonal transport. The possibility that NF-200+RGCs represent damaged cells is supported by a recent study using comparable methodology to induce OHT, in which eyes with OHT had RT97+RGCs showing typical signs of axotomy-induced neuronal degeneration [40]. Notably, the NF-200+RGC somas of the OHT-eyes in our study tended to be more abundant in areas of the retina having less GFAP-RA. It is possible that an impairment of axonal support exerted by astrocytes could increase the vulnerability of the axon to IOP-induced stress [67]. A possible explanation could be the observations reported by Dibas *et al*. [68] according to which retinas with OHT showed a downregulation of AQP4 protein and an accumulation of ubiquitin in astrocytes which might not be appropriately transferred to adjacent RGCs. The attenuation of ubiquitination in axons may result in the accumulation of several proapoptotic proteins (that is, caspases, Bax and Bad) [69] and thus contributes to axonal degeneration in glaucoma.

It is known that severe axon insult can result in a rapid Wallerian degeneration of the distal axon [70]. On the contrary, milder insults may result in degeneration via the slower process of axonal dying-back and greater functional connectivity between the soma, proximal axon, and the distal axon segments [71-76]. This situation involving the NF-200+RGCs of the present work, as reported by Soto *et al*. [56,77] could represent RGCs that have suffered an insult but retain their fundamental homeostatic mechanisms, which might provide an opportunity for therapeutic rescue in the human disease.

A striking feature of the contralateral eyes was that macroglia exhibited morphological signs of reactivity that differed from naïve and OHT-eyes: astrocytes were more robust, formed a honeycomb-like network, and had an increase in GFAP-RA. In addition, the percentage of the retinal area occupied by GFAP+ cells with intensities above 3,000 was higher than in naïve eyes but did not differ from that of OHT-eyes. By contrast, the contralateral retinas of two experimental models of glaucoma in rats exhibited a decrease in both the retinal area occupied by astrocytes [43] and the GFAP-IR in astrocytes [43,45]. The different behavior of the retinal macroglia of the contralateral eyes between mice and rats could be species related or could depend on the experimental model such as differences in time to increase pressure or time to return to normal values, among others [67].

It has been reported that the glial-activation response in glaucomatous eyes involves the activation of a glial immunoregulatory function and antigen-presenting ability [10,78]. The expression of MHC-II in glial cells, required for antigen presentation to T cells, is upregulated in the glaucomatous human retina and optic-nerve head [10,79,80]. In addition, microglial activation has been demonstrated in human eyes with glaucoma [81], in experimental models of OHT [8,80,82,83] and in a genetic mouse model of glaucoma [34]. Our study confirms the microglial activation in eyes with OHT, as evidenced by their morphological changes and stronger expression of MHC-II in most microglial cells compared to naïve eyes. Also, MHC-II immunoreaction in Müller cells supports the idea of immune activation in eyes with OHT.

A lack of relation between immune responses in DBA/2J mice retina and greater IOP has been reported [84] and recently corroborated by Bosco *et al*. [20], who saw no strict correlation between higher IOP and early microglia activation. They concluded that a rise in IOP may not be a contributory factor in these initial changes [20]. It bears mentioning that most microglial cells of the contralateral eyes (normal values of IOP) showed morphological changes and MHC-II upregulation in comparison with naïve eyes. In an experimental model of laser-induced OHT in rats, the contralateral eyes reportedly had a marginal increase in OX42 and OX6 (a MHC-II marker) two hours post-operation before returning to almost normal or normal, respectively, at three days [82]. The microglia of the contralateral retinas of the present study exhibited an upregulation of MHC-II immunoreaction that was widespread and more persistent than in the study of Wang *et al*. [82], given that in our retinas, an intense MHC-II immunoreaction of the microglia was detected throughout the retina after 15 days of lasering the treated eye. In addition, MHC-II upregulation was observed in both macroglial cell types, preferentially in astrocytes, a fact not reported by Wang *et al*. [82]. The MHC-II upregulation that we detected in the three retinal glial types of our study, which was not temporarily related to surgical eyeball manipulations, led us to postulate that an immunologically mediated process was taking place in contralateral retinas. This glial response may reflect an attempt to maintain tissue homeostasis, perhaps in an effort to protect optic axons from a compromised blood–brain barrier [20,81]. A more sustained insult or prolonged neuronal stress may lead to glial changes that could potentially contribute to neuronal decline.

In the present experiments, we did not estimate RGCs survival, but in a recent parallel study using a comparable methodology to induce OHT, we documented RGC loss using a retrograde tracer applied to both superior colliculi one week prior to animal processing. In that study, the contralateral retinas showed the typical distribution of RGCs throughout the retina [40]. Based on this data, macroglial activation as well as MHC-II expression on astrocytes and Müller cells of our contralateral eyes could have exerted a neuroprotective effect. It has been suggested that the expression of modest levels of MHC-II may inhibit the activation of invading T cell, whereas overexpression of these molecules may promote the activation of autoimmune T cells, thereby augmenting the inflammatory cascade leading to tissue damage [10,85]. This could be the case for our OHT-eyes in which GFAP-IR and MHC-II expression of Müller cells notably increased in comparison to naïve and contralateral eyes.

Another finding supporting the possible contribution of retinal macroglia to neuronal homeostasis in contralateral eyes and the harmful response of this population in OHT-eyes could be the difference between the contralateral and OHT-eyes in the NF-200+RGC count.

No previous studies are available on the expression of MHC-II on macroglia and microglia in the contralateral eye of adult Swiss mice after 15 days of laser-induced OHT. MHC-II upregulation in contralateral eyes could be secondary to IOP-induced changes in OHT-eyes. Furthermore, the absence of NF-200+RGCs (sign of RGC degeneration) leads us to postulate that the expression of modest levels of MHC-II in macroglial cells in contralateral eyes could offer a protective role.

## Conclusions

In summary, after 15 days of unilateral laser-induced OHT, widespread and persistent changes in GFAP and MHC-II expression took place both in the contralateral and OHT-eyes of adult Swiss mice. MHC-II upregulation in Iba-1+ retinal cells was similar in both eyes; however, in the retinal macroglia, MHC-II expression was preferentially located in astrocytes of the contralateral eye and appeared to be restricted to Müller cells in the OHT-eye. The increased antigen-presenting activity in macroglial and microglial cells may be key in the role of the immune system in glaucoma. Knowledge of this role could lead to the development of more effective neuroprotective treatments to modulate the immune response to achieve, on the one hand, tissue repair and neuronal survival, and on the other, a decrease in the immune-mediated neurodegenerative damage.

On the basis of the alterations in the contralateral eye, we conclude that it should not be used as a control eye in this experimental model of laser-induced OHT. Further research is needed to understand the behavior of contralateral eyes in other models of experimental OHT.

## Abbreviations

GFAP, Glial fibrillary acidic protein; GFAP-RA, GFAP-labeled retinal area; GFAP-IR, GFAP immunoreaction; Iba-1, Ionized calcium binding adaptor molecule 1; IOP, Intraocular pressure; MHC-II, Major histocompatibility complex class II molecule; NF-200, Neurofilament of 200kD; OHT, Ocular hypertension; RGCs, Retinal ganglion cells.

## Competing interests

The authors declare that they have no competing interests.

## Authors’ contributions

MSN, FJVS, AOM, MAT, MPVP and MVS carried out the development of the animal model and the IOP measurement. BIG, JJS, RdH, BR, AIR, AT, JMR contributed to the immunhistochemical study, analysis and interpretation of data, drafting the manuscript and revising it critically. All authors read and approved the final manuscript.
